# PLGA/nHA hybrid nanofiber scaffold as a nanocargo carrier of insulin for accelerating bone tissue regeneration

**DOI:** 10.1186/1556-276X-9-314

**Published:** 2014-06-25

**Authors:** Adnan Haider, Kailash Chandra Gupta, Inn-Kyu Kang

**Affiliations:** 1 Department of Polymer Science and Engineering, School of Applied Chemical Engineering Kyungpook, National University, Daegu 702-701, South Korea; 2 Polymer Research Laboratory, Department of Chemistry, I.I.T., Roorkee 247667, India

**Keywords:** Hydroxyapatite, PLGA, Insulin

## Abstract

The development of tissue engineering in the field of orthopedic surgery is booming. Two fields of research in particular have emerged: approaches for tailoring the surface properties of implantable materials with osteoinductive factors as well as evaluation of the response of osteogenic cells to these fabricated implanted materials (hybrid material). In the present study, we chemically grafted insulin onto the surface of hydroxyapatite nanorods (nHA). The insulin-grafted nHAs (nHA-I) were dispersed into poly(lactide-co-glycolide) (PLGA) polymer solution, which was electrospun to prepare PLGA/nHA-I composite nanofiber scaffolds. The morphology of the electrospun nanofiber scaffolds was assessed by field emission scanning electron microscopy (FESEM). After extensive characterization of the PLGA/nHA-I and PLGA/nHA composite nanofiber scaffolds by Fourier transform infrared spectroscopy (FTIR), X-ray diffraction spectroscopy (XRD), X-ray photoelectron spectroscopy (XPS), energy-dispersive X-ray spectrometry (EDS), and transmission electron microscopy (TEM), the PLGA/nHA-I and PLGA/nHA (used as control) composite nanofiber scaffolds were subjected to cell studies. The results obtained from cell adhesion, alizarin red staining, and Von Kossa assay suggested that the PLGA/nHA-I composite nanofiber scaffold has enhanced osteoblastic cell growth, as more cells were proliferated and differentiated. The fact that insulin enhanced osteoblastic cell proliferation will open new possibilities for the development of artificial scaffolds for bone tissue regeneration.

## Background

Polymeric fibers have been fabricated using various techniques such as self-assembly, phase separation, melt spinning, and electrospinning. Among these, electrospinning is a unique, simple, cost-effective, versatile, and scalable technique used for the fabrication of nanofibers from a wide range of natural and synthetic polymers [1-4]. Electrospinning is used frequently in the engineering, environmental, and biomedical fields [5,6]. Fibrous scaffolds prepared via electrospinning exhibit unique properties such as a high surface area-to-volume ratio, ultrafine uniform fibers, having high porosity and variable pore size distribution within the intra-fibrous structure [4]. These properties serve to enhance the biocompatibility and biological responses of the scaffold. The electrospinning process is quite flexible; therefore, it provides more efficient control over the nanofiber scaffold by altering several governing experimental parameters, including polymer concentration, voltage, and needle to collector distance [7]. Until now, a variety of synthetic as well as natural biopolymers have been used to date for the preparation of fibrous scaffolds by electrospinning [8,9]. Among synthetic polymers, poly(lactide-co-glycolide) (PLGA), a biodegradable polyester, has been studied extensively in the preparation of electrospun scaffolds. Apart from biocompatibility, PLGA exhibits excellent biodegradability over time and its degradation rate can be altered by adjusting the monomer ratio [10,11]. A series of experiments have concluded favorable cellular responses to these nanofibrous scaffolds; Kim et al. demonstrated enhanced osteoblast adhesion and proliferation onto electrospun nanofiber scaffolds [1].

Inorganic nanomaterials such as nanotubes, nanocrystals, nanorods, nanospheres, nanoparticles, and nanofibers have unique properties, which cannot be achieved by using pristine polymers. During the electrospinning process, several inorganic fillers, including β-tricalcium phosphate (β-TCP), hydroxyapatite nanorods (nHA), multiwall carbon nanotubes (MWCNT), and calcium carbonate (n-CaCO_3_) are successfully incorporated into the polymer solution to fabricate biocomposite electrospun scaffolds for tissue engineering [1]. HA is among one of the widely used bioceramic material having similar composition and morphology to the inorganic component of natural bone [12]. In addition, it can provide a favorable environment for cell adhesion, osteoconduction, and osteoinduction.

Controlling the surface energies enables us to precisely control the surface and interfacial properties of nanomaterials ranging from wetting to adhesion, thus providing an active site for chemical reactions and/or interactions with foreign bodies. This can be achieved by tailoring the surface of nanomaterials [2,13]. Recently, several reports have described strategies for surface modification, including the chemical attachment of long or short-chain molecules to a wide range of surfaces or substrates [14,15]. Succinic acid is used as a surface modifier and carrier for targeted drug delivery systems (DDS) on nanomaterial surfaces due to its non-immunogenic, non-toxic, and non-antigenic properties [16]. Succinic acid can alter the physical and chemical properties of the substrates [17], where the substrate surfaces modified by succinic acid are more prone to chemical reactions with suitable functional groups such as the primary amine group (NH_2_). The functional groups provide active sites for the covalent conjugation of the protein with other macro- and micromolecules and hence improve the biocompatibility and dispersion properties of the substrate. The incorporation of bioactive agents by mixing, encapsulation, or covalent bonding to electrospun fibers could lead to advanced biofunctional tissue engineering (TE) scaffolds [18]. The biofunctionalization of electrospun fibers is, however, the most prominent method used and determines the efficiency of these fibers to regenerate biofunctional tissues. Insulin is a peptide protein capable of regulating carbohydrate and fat metabolism in the body [19]. It is highly effective in controlling diabetes mellitus and is used in the treatment of diabetes [20]. In addition, insulin is a well-known cell growth factor capable of enhancing cell proliferation, including activation of muscle stem cells [20-22]. Therefore, several insulin-like growth factors were used previously in the field of bone regeneration, which showed high biocompatibility and enhanced cell growth [23].

The aim of the present study was to enhance the cell affinity, osteoconduction, and osteoinduction by grafting insulin onto the surface of nHA by chemical reaction, which was used to fabricate three-dimensional electrospun PLGA/nHA-I composite nanofiber scaffolds. The adhesion, proliferation, and differentiation of MC3T3 cells were investigated to evaluate the potential of the PLGA/insulin-grafted nHAs (nHA-I) nanofiber composite as a bone TE scaffold.

## Methods

PLGA (lactide/glycolide 85:15), with molecular weight of 240,000, insulin from the human pancreas, and succinic acid were purchased from Sigma-Aldrich (St. Louis, MO, USA). nHA was synthesized in the laboratory. Minimal essential medium (MEM)-alpha and the osteoblast MC3T3-E1 cell line were purchased from the Korea cell bank (Seoul, South Korea). 5-Bromo-2-deoxyuridine (Brdu) and alizarin red staining kits were purchased from Roche Molecular Biochemicals (Indianapolis, IN, USA) and Millipore (Billerica, MA, USA), respectively. Fetal bovine serum (FBS) and penicillin G-streptomycin were purchased from Gibco, Tokyo, Japan. All reagents and chemicals in this study were used without any further purification.

### Synthesis of nHA

nHA was synthesized via chemical precipitation, as previously described [24]. Briefly, 400 ml (NH_4_)_2_PO_3_ and 300 ml CaNO_3_ · 4H_2_O solutions were prepared separately by dissolving 19.75 g (NH_4_)_2_PO_3_ and 57.5 g (CaNO_3_) · 4H_2_O in distilled water. The pH of (CaNO_3_) · 4H_2_O solution was adjusted to 10.4 with NH_4_OH, after which the two solutions were mixed dropwise with vigorous stirring. During mixing, a white precipitate was formed, which was aged for 4 days to form nHA. The synthesized nHA was washed with distilled water until the pH reached 7. The nHA was resuspended in 1-butanol to prevent nHA from aggregation during the drying process. Finally, the precipitate was dried at 80°C and calcined at 500°C for 4 h to remove rudimental organic compounds.

### Surface grafting of nHA via insulin

**Figure 1:**
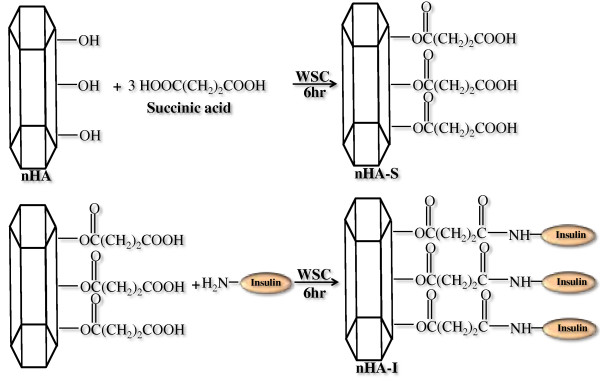
Schematic diagram depicting grafting of insulin on the surface of nHA.

The grafting of insulin on the surface of nHA was carried out in two steps. First, the carboxyl group (-COOH) was introduced onto the nHA surface via a reaction between succinic acid and surface hydroxyl groups of nHA. Subsequently, in the second step, insulin was grafted onto the surface of succinic acid-modified nHA via chemical reaction, between the amine group (NH_2_) of insulin and the free terminal carboxylic group (COOH) of succinic acid using water-soluble carbodimide (WSC). Briefly, an excess amount of succinic acid was dissolved in distilled water (DI). Then, the free carboxylic acid groups of succinic acid were activated using WSC and kept for 6 h at room temperature with gentle stirring to activate the terminal carboxylic groups. After this activation step, nHA was added to the aqueous solution of succinic acid and 1-ethyl-3-(3-dimethylaminopropyl) carbodiimide hydrochloride (EDC, 0.5 g; 0.25 wt.%) and *N*-hydroxysuccinimide (NHS, 0.05 g, 0.25 wt.% ) and kept for 6 h with constant, gentle stirring. The succinic acid-grafted nHA (nHA-s) were washed twice with double distilled water, centrifuged at 13,000 rpm, and freeze-dried. In the second step, the nHA-s were resuspended in an aqueous solution containing WSC solution and stirred gently for 6 h at room temperature in order to activate the free terminal (COOH) group. This was followed by addition of an equal amount of insulin corresponding to the amount of nHA-s. The solution was stirred gently for 12 h at room temperature to obtain nHA-I (Figure 1). The nHA-I was then washed with distilled water to remove impurities and freeze-dried.

### Solution preparation and electrospinning

PLGA polymer solution in the concentration range of 5 to 20 wt.%, was prepared by dissolving in a binary solvent (THF and DMF in a 3:1 ratio). The solution was stirred overnight at room temperature until complete dissolution. The solution was then subjected to electrospinning. For this, the PLGA solution was placed into a 10-mL glass syringe fitted with a needle of 0.9 mm (20 G) inner diameter. A typical electrospinning setup consists of four main components: (i) a pump, to hold and pump the hypodermic syringe containing polymer solution, which allowed controlled outflow of the polymer solution; (ii) a high voltage supply of 1 to 50 kV; (iii) a metallic capillary (needle) connecting the syringe to the positive voltage; and (iv) a metallic collector (flat or rotating drum), which can either be stationary or rotating) connected to negative voltage. The electrospinning process began when a high electric current was generated from the power supply. The solution moved to the tip of the needle, and the hemispherical shape of the droplet was destabilized by charges that accumulated on its surface. As the charges balanced the fluid surface tension of the polymer solution, the droplet was converted to a Taylor's cone with a semivertical angle of approximately 30° [25]. At a critical electrical voltage, the electric forces surpassed the surface tension of the droplet and a jet of ultrafine fibers emanated from the tip of the Taylor's cone and was collected onto the collector kept at fixed distance [26]. Due to the high electric voltage used in the process, the fluid jet usually remains stable for a small distance (2 to 4 cm) before scattering. The optimized electrospinning conditions used in the present study were tip-to-collector distance 20 cm, applied voltage 20 kV, needle diameter 20 G (0.9 mm), and flow rate 1 mL/h. The electrospun nanofibers collected were removed from the collector and dried overnight at 40°C to remove the remaining solvent. After drying, the sample was sputter-coated with gold and its morphology was observed by field emission scanning electron microscopy (FESEM; 400 Hitachi, Tokyo, Japan). The same procedure was adapted for the preparation of the electrospun PLGA/nHA-I and PLGA/nHA composite nanofiber scaffolds. Briefly, both pristine nHA and insulin-grafted nHA-I were added into the PLGA polymer solution and were mechanically dispersed via alternate stirring and sonication. After dispersion, the samples were subjected to electrospinning process.

### Osteoblastic cell culture

To examine the interaction of the PLGA/nHA-I and PLGA/nHA composite nanofiber scaffolds with osteoblastic cells (MC3T3-E1), the composite nanofiber scaffolds were cut into small circular discs, fitted inside a 4-well culture dish, and immersed in MEM medium containing 10% FBS (Gibco; Invitrogen, Carlsbad, CA, USA). Subsequently, 1 mL of the MC3T3-E1 cell solution (3 × 10^4^ cells/mL) was added to the surface of the composite nanofiber scaffolds and incubated in a humidified atmosphere containing 5% CO_2_ at 37°C for 1 and 3 days. After incubation, the supernatant was removed and the composite nanofiber scaffolds were washed twice with phosphate-buffered saline (PBS; Gibco, Langley, OK, USA) and fixed in a 2.5% glutaraldehyde solution for 15 min. The samples were then dehydrated, dried in a critical point drier, and sputter-coated with gold. The surface morphology of the composite nanofiber scaffolds was observed by FESEM (400 Hitachi; Tokyo, Japan).

### Cytoskeletal organization

To evaluate the cytoskeletal organization of cells onto the PLGA/nHA-I and PLGA/nHA composite as well as pristine PLGA nanofiber scaffolds, double staining was performed according to the manufacturer's protocol. Briefly, osteoblast cells were seeded onto the scaffolds (2 × 10^4^ cells/mL) and were cultured for 3 days. The cells were fixed with 4% paraformaldehyde in PBS. After fixation, the samples were washed using PBS buffer solution containing (0.05% Tween-20). The samples were permeabilized with 0.1% Triton X-100 in PBS for 15 min at 25°C and then incubated for 30 min in PBS containing 1% bovine serum albumin (BSA). This was followed by the addition of 5(6)-tetramethyl-rhodamine isothiocyanate-conjugated phalloidin (Millipore) (TRITC) for approximately 1 h. The samples were washed three times (10 min each) using the buffer solution and incubated with 4′,6-diamidino-2-phenylindole (DAPI) (Millipore) for 5 min. Fluorescence images were visualized using a confocal laser scanning microscope (model 700; Carl Zeiss, Oberkochen, Germany) after washing the scaffolds three times (10 min each) with the buffer solution.

### Cell proliferation

Proliferation of MC3T3 osteoblastic cells seeded on the PLGA/nHA-I, PLGA/nHA composite, and pristine PLGA nanofiber scaffolds was determined using a colorimetric immune assay, based on the measurement of BrdU, which was incorporated during DNA synthesis. BrdU enzyme-linked immunosorbent assay (ELISA; Roche Molecular Biochemicals) was performed according to the manufacturer's instructions. Briefly, after cell culture for 48 h, BrdU-labeling solution was added to each well. The solution was allowed to incorporate into the cells in a CO_2_ incubator at 37°C for 20 h. Subsequently, the supernatant in each well was removed by pipetting and washed twice with PBS. The cells were treated with 0.25% trypsin-ethylenediaminetetraacetic acid (EDTA) (Gibco, Tokyo, Japan) and harvested by centrifugation of the cell solution at 1,000 rpm for 15 min. The harvested cells were mixed with FixDenat solution to fix the cells and denature the DNA and then incubated for 30 min. Subsequently, diluted anti-BrdU peroxidase (dilution ratio of 1:100) was added to the cells and incubated at 20°C for 120 min. After removing the unbound antibody conjugate, 100 μL substrate was added and allowed to stand for 20 min. The reaction was completed by adding 25 μL H_2_SO_4_ solution (1 M). The solution was then transferred to a 96-well plate and measured within 5 min at 450 nm with a reference wavelength of 690 nm, using an ELISA plate reader (EL 9800). The blank reading corresponded to 100 μL of culture medium with or without BrdU.

### Alizarin red staining

Alizarin red staining of the MC3T3 osteoblastic cells cultured on the electrospun PLGA/nHA-I, PLGA/nHA, and pristine PLGA nanofiber scaffolds was performed to examine mineralization and differentiation. Briefly, after culturing the MC3T3 osteoblasts, the medium was aspirated without disturbing the cells. The culture dish with the osteoblastic cells was washed twice with PBS. The cells were then fixed with 10% formaldehyde and incubated for 15 min at room temperature. The fixative reagent was removed carefully, and the cells were rinsed three times (10 min each) with distilled water to avoid disturbing the monolayer. After washing, the excess water was removed and alizarin red staining solution (1 mL/well) was added to the cells and the samples were incubated for 30 min. Subsequently, the excess amount of dye was removed from the stained cells by washing the samples four times with distilled water (5 min each) with gentle rocking. Digital images of the stained cells were obtained with a camera (Nikon E 4500, Tokyo, Japan).

### Von Kossa assay

Calcium deposition of MC3T3-E1 cells was examined by Von Kossa staining. The cells were cultured for 15 days on PLGA/nHA-I, PLGA/nHA, and pristine nanofiber scaffolds under the same conditions as those described in the alizarin red staining experiment. After incubation, the cells were washed three times with PBS for 5 min, fixed with 10% formaldehyde for 30 min, and washed three times with distilled water for 10 min. The fixed samples were treated with 5% AgNO_3_ solution for 5 min under ultraviolet radiation. After removing the AgNO_3_ solution, the samples were washed with PBS twice followed by the addition of 5% Na_2_S_2_O_3_ solution to the plate and allowing the plates to stand for 5 min. Finally, the samples were washed twice with distilled water and digital images of the stained cells were obtained.

### Statistical analysis

The results are displayed as the mean ± standard deviation. The statistical differences were determined using a student's two-tailed test. Scheffe's method was used for the multiple comparison tests at a level of 95%.

## Results and discussion

### Preparation of nanofiber scaffolds

**Figure 2:**
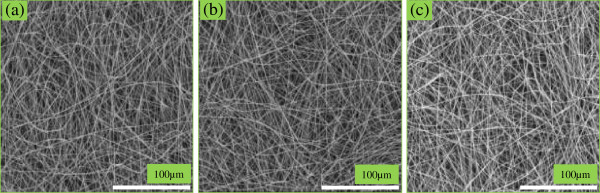
FESEM images of (a) pristine PLGA, (b) PLGA/nHA, and (c) PLGA/nHA-I nanofiber scaffolds.

Figure 2 illustrates the FESEM images of the electrospun PLGA/nHA-I, PLGA/nHA, and pristine PLGA nanofibers scaffolds. With optimized electrospinning parameters, no remarkable change was observed in the morphology of pristine PLGA, PLGA/nHA, or PLGA/nHA-I composite nanofiber scaffolds. The nanofibers were smooth and beadless in all the samples. However, the average diameters of PLGA/nHA (mean average diameter 500 nm) and PLGA/nHA-I (mean average diameter 520 nm) composite nanofibers increased slightly as compared to pristine PLGA nanofiber having (mean average diameter 450 nm). This increase in the average diameter might be due to the incorporation of pristine nHA and nHA-I in the PLGA polymer matrix. A similar increase in the average diameter of the modified nanofibers has been also reported elsewhere [27].

### Fourier transform infrared spectroscopy study

**Figure 3:**
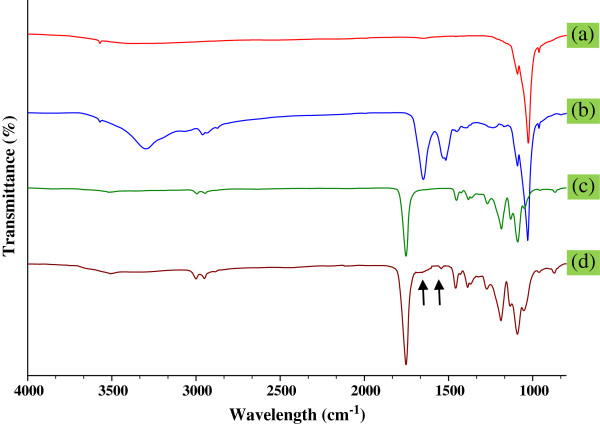
FTIR spectra of (a) pristine nHA, (b) nHA-I, (c) pristine PLGA, and (d) PLGA/nHA-I.

Figure 3 illustrates the Fourier transform infrared (FTIR) spectra of the pristine nHA, nHA-I, pristine PLGA, and PLGA/nHA-I composite nanofiber scaffolds. The sharp band, which appeared in the regions of 1,000 to 1,100 cm^-1^ in the pristine nHA spectrum is characteristic of a regular tetrahedral (PO_4_^-3^) of nHA (Figure 3(a)) [28,29]. The appearance of weak doublet bands in the region of 2,800 cm^-1^ to 3,200 cm^-1^ in nHA-I spectrum (Figure 3(b)) was attributed to hydrocarbons (CH, CH_2_) of succinic acid [30]. The two sharp bands at 1,648 and 1,540 cm^-1^ were attributed to the stretching vibration of the carbonyl group (C = O) within amide I (-CO-NH) and the coupling of N-H bending and C-N stretching of amide II (-CO-NH) [31]. The appearance of these bands at their characteristic positions confirmed the grafting insulin on the surface of succinic acid-modified nHA-s. The band at 3,500 cm^-1^ was attributed to the free carboxylic acid (COOH) moiety present in insulin [28]. A sharp peak at 1,742 cm^-1^ appeared in the PLGA polymer spectrum (Figure 3(c)), which was assigned to the C = O stretching of PLGA polymers. Similarly, the bands at 2,800 to 3,200 cm^-1^ were assigned to hydrocarbons (CH, CH_2_) [30]. In the spectrum of the PLGA/nHA-I (Figure 3(d)), all the abovementioned bands were present at their characteristic positions. However, the reduced intensities of the bands for amide and carboxylic functionalities might be attributed to the influence of the excess amount of PLGA used.

### X-ray photoelectron spectroscopy analysis

**Figure 4:**
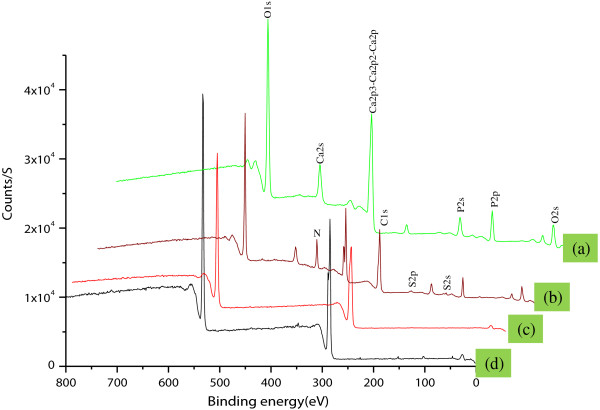
XPS graph of (a) pristine nHA, (b) nHA-I, (c) pristine PLGA nanofiber scaffold, and (d) PLGA/nHA-I nanofiber composite scaffolds.

The successful grafting of insulin on nHA using succinic acid as a spacer was confirmed by X-ray spectroscopy (XPS) (ESCA). Figure 4 shows the data obtained from the qualitative analysis of pristine nHA, nHA-I, PLGA, and PLGA/nHA-I. The N1s and S2p photoelectron signals were the markers of choice for confirmation of insulin grafting on the surface of succinic acid-modified nHA-s and the presence of insulin-grafted nHA-I in the PLGA nanofibers. nHA showed three photoelectron signals (Figure 4(a)), corresponding to Ca2p (347.9 eV) and O1s (binding energy 536.1 eV) along with P2p (binding energy, 133.2 eV). Whereas PLGA (Figure 4(c)) showed two photoelectron signals, representing C1s (binding energy, 284.6 eV) and O1s (binding energy, 536.1 eV). On the other hand, two new photoelectron signals were observed for the PLGA/nHA-I composite (Figure 4(d)) and nHA-I (Figure 4(b)), namely, representing nitrogen (N1s, at binding energy 397.9 eV) and sulfur (S2p, binding energy 164.05 eV), respectively. This confirmed successful grafting of insulin on the surface of pristine nHA Figure 4(b), and the presence of insulin-grafted nHA-I in the PLGA composite nanofiber scaffold PLGA polymer (Figure 4(d)).

Table 1 shows that the atomic wt.% of nitrogen (N) and sulfur (S) was zero in pristine nHA and PLGA. However, when the surface of nHA was modified with succinic acid and subsequently on grafting with insulin, the atomic wt.% of calcium (Ca) and phosphorous (P) decreased, whereas those of carbon (C), nitrogen (N), and sulfur (S) increased due to succinic acid and further grafting of insulin on the surface of nHA. This increase in atomic wt.% clearly indicated that succinic acid and insulin had been successfully grafted onto pristine nHA. Through the addition of nHA-I to PLGA, the atomic wt.% of calcium (Ca), phosphorous (P), nirtogen (N), and sulfur (S) decreased whereas the atomic wt.% of carbon (C) increased, confirming the presence of nHA-I in the PLGA nanofiber matrix.

**Table 1:** Chemical composition of nanofiber scaffolds calculated from ESCA (XPS) survey scan spectra

**Substances**	**Atomic weight (%)**					
	**C**	**0**	**Ca**	**N**	**P**	**S**
nHA	7.7	66.6	17.8		12.6	
PLGA	64.61	35.39				
nHA-I	47.77	30.90	11.51	6.75	5.2	0.76
PLGA/nHA-I	63.38	27.40	4.12	3.10	2.75	0.25

### X-ray diffraction spectroscopy study

**Figure 5:**
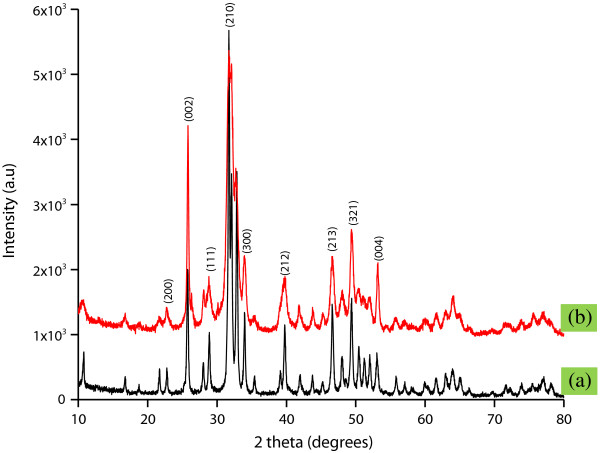
XRD profile of (a) pristine nHA and (b) nHA-I.

Figure 5 depicts the X-ray diffraction spectroscopy (XRD) profile of pristine nHA and nHA-I. From the XRD profile, it is evident that grafting of insulin had no adverse effect on the crystallinity of nHA. The characteristic diffraction peaks of nHA before and after grafting of insulin appeared at 26.1°, 28.45°, 30.1°, 32.90°, 35.97°, 40.19°, 41.82°, 53.56°, 55.75°, 57.40°, 69.12°, 74.45°, and 77.56°, corresponding to the 002, 102, 210, 112, 300, 212, 130, 213, 321, 004, and 104 planes, respectively, of the nHA unit cell with hexagonal symmetry. The peaks were at the same positions in both pristine nHA and nHA-I [28]. From the XRD profile of pristine nHA and nHA-I, it was found that the crystallinity of the nHA was intact even after grafting with insulin.

### Transmission electron microscopy (TEM) morphology study

**Figure 6:**
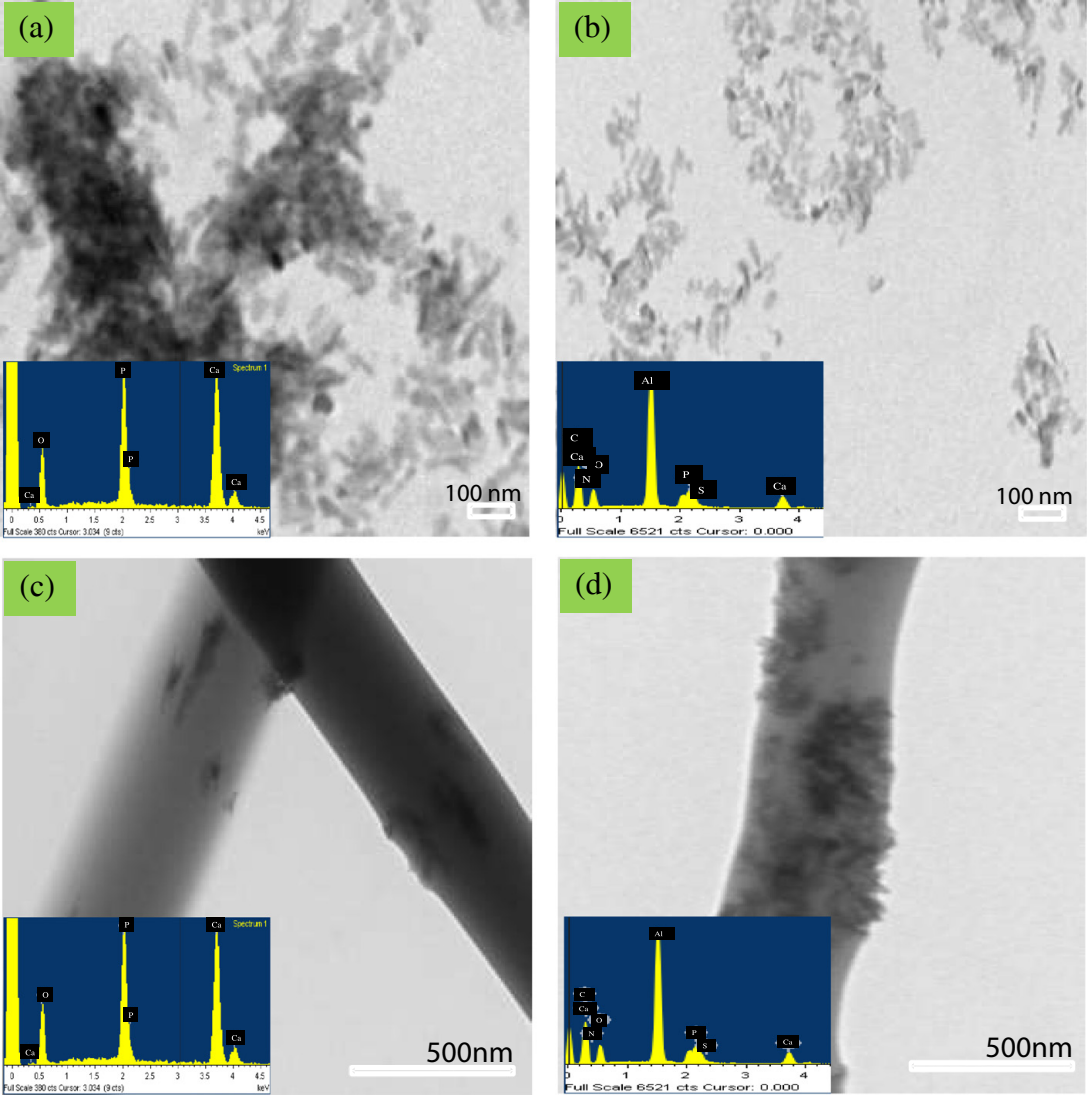
**TEM images of (a) pristine nHA, (b) nHA-I, (c) PLGA/nHA, and (d) PLGA/nHA-I with their respective EDX graphs.** Depicting their characteristics peaks and chemical compositions.

**Figure 7:**
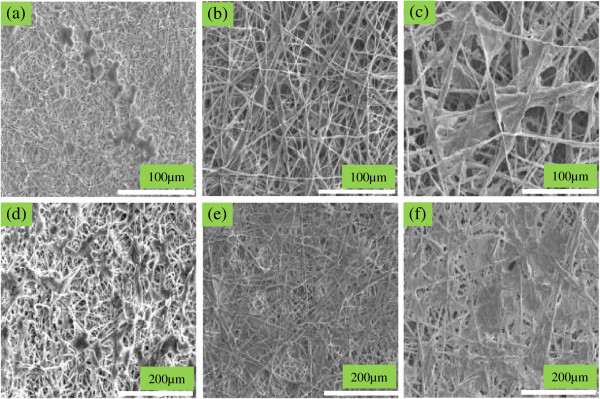
**SEM images of the osteoblast adhesion on (a, d) pristine PLGA, (b, e) PLGA/nHA, (c, f) PLGA/nHA-I.** After 1 day **(a, b, c)** and 3 days **(d, e, f)** of incubation.

The morphology of pristine nHA and nHA-I embedded in the PLGA matrix was observed under TEM. Figure 6a,b illustrates the TEM images of pristine nHA and nHA-I. From the TEM images, it is obvious that nHA-I (Figure 6b) was well dispersed as compared to pristine nHA (Figure 6a), which formed agglomerated clusters on the hydrophobic carbon grid. Dispersion of nHA in the PLGA nanofiber scaffold was improved by the incorporation of insulin to the nHA (nHA-I) as compared to the pristine and grafted nHA. Energy-dispersive X-ray spectroscopy (EDX) data in the downset of Figure 6a,b show the characteristic peaks of calcium (Ca), phosphorus (P), and oxygen (O) for pristine nHA (Figure 6a) and calcium (Ca), phosphorus (P), nitrogen (N), and sulfur (S) for nHA-I (Figure 6b). The presence of these peaks endorsed that the dispersed materials were pristine nHA and nHA-I. Furthermore, characteristic EDX peaks of pristine nHA and nHA-I were also observed for PLGA/nHA (Figure 6c) and PLGA/nHA-I composite nanofiber scaffolds (Figure 6d). This confirms the presence of nHA and nHA-I in PLGA/nHA (Figure 6c) and PLGA/nHA-I composite nanofiber scaffolds (Figure 6d). Figure 6c,d depicts the morphology of the composite nanofibers. The composite nanofibers were uniform, with pristine nHA and nHA-I embedded in the PLGA electrospun nanofibers. Because of its hydrophilic nature, pristine nHA showed restricted dispersion in the hydrophobic PLGA polymer (Figure 6c) [32]. However, on the other hand, grafting of insulin on the surface of pristine nHA enhanced the dispersion of nHA in the PLGA polymer matrix (Figure 6d) [33]. The relatively uniform dispersion of nHA-I in the PLGA polymer matrix was beneficial for the osteoblastic cell adhesion analysis because one portion of the nHA-I was embedded while the rest protruded from the electrospun PLGA/nHA-I composite nanofibers surface. The protrusion of nHA-I made the surface of the PLGA/nHA-I rough. Thus, more cells were able to adhere to the rough surface of PLGA/nHA-I and proliferate, in contrast to the smooth surface of PLGA/nHA and PLGA nanofibers (Figure 7) [34]. The protrusion of nHA-I originated due to the phase separation between the hydrophilic nHA-I and the hydrophobic PLGA polymer matrix [35].

### Bioactivity and cellular response

The adhesion behavior of the osteoblastic cells to implantable materials is determined mostly by their surface chemistry and topography [36]. To elucidate the *in vitro* osteoblastic cell behavior and assess the effectiveness of insulin grafting onto the surface of nHA, osteoblastic cells were cultured on pristine PLGA nanofiber scaffolds as well as PLGA/nHA and PLGA/nHA-I composite nanofiber scaffolds. As depicted in Figure 7, more cells adhered to the PLGA/nHA-I composite nanofiber scaffolds (Figure 7c,f) contrary to the PLGA/nHA composite (Figure 7b,e) and pristine PLGA nanofiber scaffolds (Figure 7a,d). The increased adhesion of osteoblastic cells to PLGA/nHA-I composite nanofiber scaffolds was attributed to the presence of nHA-I in the PLGA nanofiber scaffold (PLGA/nHA-I) and to the rough morphology of the PLGA/nHA-I composite nanofiber scaffolds due to the protrusion of the nHA-I from the PLGA nanofiber scaffolds (Figure 6d). Insulin has the capability of enhancing cell growth [20,22], whereas protrusion makes the surface of the scaffold rough. Osteoblastic cells adhesion was enhanced in both cases [20,22,34,36]. The order of increase in cell adhesion and spreading of osteoblastic cells was PLGA/nHA-I > PLGA/nHA > PLGA. Besides the type of scaffolds, adhesion of the osteoblastic cells was also increased with an increase in incubation time from 1 to 3 days. In addition to better adhesion, more spreading of osteoblastic cells was observed on the PLGA/nHA-I composite nanofiber scaffold as compared to the PLGA/nHA composite and pristine PLGA nanofiber scaffolds.

**Figure 8:**
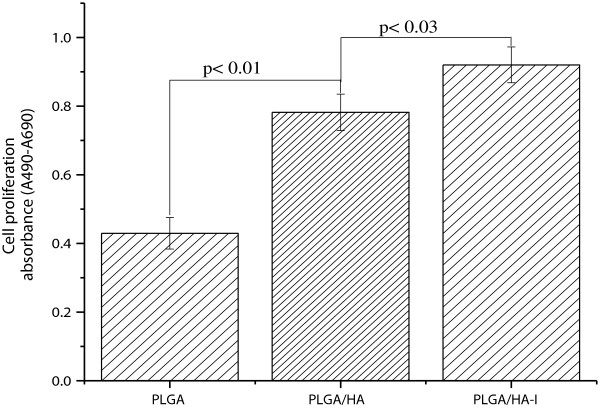
**Proliferation of osteoblast cells cultured on the pristine PLGA, PLGA/nHA, and PLGA/nHA-I nanofiber scaffolds.** For 2 days as determined by a Brdu assay.

**Figure 9:**
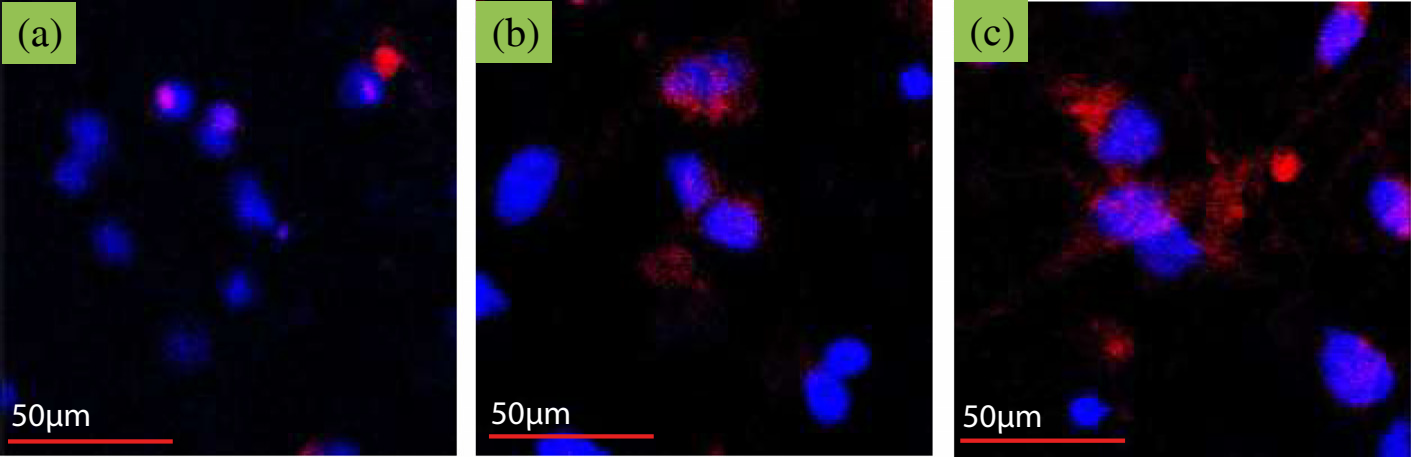
**Confocal laser scanning micrograph of osteoblasts. Actin (red). Nucleus (blue). (a)** Pristine PLGA, **(b)** PLGA/nHA, and **(c)** PLGA/nHA-I after 3 days of incubation.

Figure 8 represents the results obtained from the Brdu assay after culturing osteoblastic cells on pristine PLGA, PLGA/nHA, and PLGA/nHA-I composite nanofiber scaffolds. The proliferation of the osteoblastic cells on the PLGA/nHA-I composite nanofiber scaffold was better as compared to the PLGA/nHA composite and pristine PLGA nanofiber scaffolds. This was attributed to the widely accepted role of insulin as a cell growth factor [21]. These results indicated that insulin played a vital role in stimulating growth and proliferation of mature osteoblastic cells by enhancing the biocompatibility of the PLGA/nHA-I composite nanofiber scaffold. Thus, more osteoblastic cells proliferated on the PLGA/nHA-I composite nanofiber scaffold as compared to the PLGA/nHA composite and pristine PLGA nanofiber scaffolds.Figure 9 represents confocal laser microscope images of the nucleus and actin cytoskeleton staining of the osteoblastic cells cultured on pristine PLGA, PLGA/nHA, and PLGA/nHA-I composite nanofiber scaffolds for 3 days. The actin microfilament cytoskeleton is involved in cellular processes, determining cell shape, and cell attachment. As the cell adheres to a substrate material, filopodia are formed. They are moved into place by actin acting upon the plasma membrane. Our results showed that the degree of cytoskeletal organization strongly increased on PLGA/nHA-I nanofiber scaffolds (Figure 9c) contrary to the PLGA/nHA composite (Figure 9b) and pristine PLGA nanofiber scaffolds (Figure 9a). The organized cytoskeleton can exert forces onto the substratum, thus orientating the matrix. This ordered extracellular matrix can in turn orientate with the cytoskeleton of other cells that come into contact with it, ultimately creating a large-scale organization.

### Alizarin red staining

**Figure 10:**
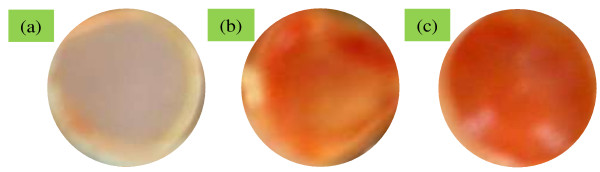
**Alizarin red staining of osteoblast cells cultured for 15 days.** On **(a)** PLGA, **(b)** PLGA/nHA, and **(c)** PLGA/nHA-I nanofiber scaffolds.

Differentiation of osteoblastic cells is one of the most important parameters for confirming osteogenesis of osteoblastic cells cultured on the scaffolds [37]. To confirm osteogenesis, alizarin red staining is considered as one of the marker specific for differentiation of osteoblastic cells [38]. Figure 10a,b,c shows that osteoblastic cells underwent osteogenesis process on all of the scaffolds. The osteogenesis process was determined from the appearance of the red color, which is an indicator of calcium production by osteoblastic cells. More cells were differentiated on the PLGA/nHA-I composite nanofiber scaffold (Figure 10c, dark red color) compared to the PLGA/nHA composite (Figure 10b, light red color) and pristine PLGA (Figure 10a, grayish color) nanofiber scaffolds. These results suggest that grafting of insulin on the nHA surface accelerated the differentiation of osteoblastic cells [38].

### Von Kossa assay

**Figure 11:**
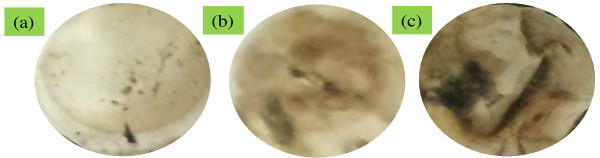
**Von Kossa assay of the osteoblast cells.** On the **(a)** PLGA, **(b)** PLGA/nHA, and **(c)** PLGA/nHA-I scaffolds after 15 days of incubation.

Figure 11 illustrates the results of the Von Kossa assay performed on the PLGA/nHA-I, PLGA/nHA composite, and pristine PLGA nanofiber scaffolds. Bone nodules are considered to be one of the markers specific to osteoblastic cell differentiation. In the Von Kossa assay, the calcified area is stained as black spot. The results obtained from the Von Kossa assay suggest that more bone nodules were formed on the PLGA/nHA-I (Figure 11c) contrary to the PLGA/nHA (Figure 11b) composite and pristine PLGA (Figure 11a) nanofiber scaffolds [1]. The Von Kossa assay results clearly suggested that insulin triggered and accelerated osteoblastic cell differentiation (Figure 11c) [20]. The high calcification area obtained from PLGA/nHA-I by the assessment of the scaffolds using Von Kossa assay was well matched with the dark red color obtained from PLGA/nHA-I using alizarin red staining as depicted in Figure 10c. Furthermore, PLGA/nHA composite nanofiber scaffolds showed enhanced cell differentiation (Figure 10b and 11b) due to the nHA effect as compared to the pristine PLGA nanofiber scaffolds (Figure 10a and 11a). The order of osteoblastic cell differentiation of the scaffolds was pristine PLGA < PLGA/nHA < PLGA/nHA-I [24].

## Conclusions

Insulin was grafted on the surface of hydroxyapatite nanorods to produce surface-modified (nHA-I) composite nanofiber scaffolds, composed of PLGA and nHA-I obtained by blending of nHA-I with PLGA and subsequent electrospinning. After confirming the presence of nHA-I in the PLGA matrix, the scaffolds were subjected to the cell culture studies for assessing their biocompatibility and bioactivity. The results obtained from the *in vitro* studies indicate that the cell adhesion, proliferation, and differentiation of the osteoblastic cells were accelerated on PLGA/nHA-I composite nanofiber scaffold as compared to PLGA/nHA composite and pristine PLGA nanofiber scaffolds. This study will prove a potential step forward in triggering research on bone tissue engineering, bone remodeling, artificial bone implantation, and site-specific drug delivery for various bone diseases.

## Competing interest

The authors declare that they have no competing interests.

## Authors' contributions

AH performed all the experiment, analyzed the experimental data, and drafted the manuscript. KCG helped in assessing the spectroscopic analysis. IKK conceived the study and participated in its design and in refining the manuscript and coordination. All authors read and approved the final manuscript.
